# Development and validation of a Malay version questionnaire for assessing risk perception of type 2 diabetes (RPDM)

**DOI:** 10.1371/journal.pone.0311834

**Published:** 2025-01-07

**Authors:** Miaw Yn Jane Ling, Norfazilah Ahmad, Mohd Firdaus Mohd Radi, Azimatun Noor Aizuddin

**Affiliations:** 1 Department of Public Health Medicine, Faculty of Medicine, Universiti Kebangsaan Malaysia, Kuala Lumpur, Malaysia; 2 Medical Development Division, Ministry of Health, Putrajaya, Malaysia; Tabriz University of Medical Sciences, ISLAMIC REPUBLIC OF IRAN

## Abstract

**Background:**

The burden of type 2 diabetes is increasing globally. Risk perception of type 2 diabetes plays an important role in motivating adoption of healthy lifestyle and preventive health interventions. To address the increasing burden of type 2 diabetes in Malaysia, a better understanding on its risk perception is needed as a guide for preventive interventions. This study was aimed at developing and validating a Malay-language questionnaire to assess the risk perception of type 2 diabetes (RPDM) among Malaysians.

**Methods:**

The questionnaire (RPDM) was developed based on the Health Belief Model and previous literature. A 59-item question pool was initially developed, reviewed by experts for content validity and pretested on five respondents. Different samples were used for pilot study (n = 100) and subsequent validation study (n = 158). Exploratory factor analysis (EFA) and Confirmatory factor analysis (CFA) were used to evaluate construct validity and construct reliability of the questionnaire.

**Results:**

The EFA yielded five-factors model, including 48 items with good factor loadings of > 0.40. CFA was carried out using item parceling. The final model shows acceptable model fit and had sufficient convergent and discriminant validity. The value of construct reliability ranged between 0.76 and 0.90.

**Conclusion:**

This newly developed and validated Malay-language RPDM questionnaire is valid and reliable for measuring the five constructs (perceived self-efficacy, perceived severity, perceived benefit, perceived susceptibility and perceived barrier) of risk perception of type 2 diabetes among Malaysians. This Malay-language questionnaire may contribute to a better understanding of risk perception of type 2 diabetes among Malaysians, as well as enhancement of diabetes prevention communication between healthcare providers and their clients.

## Introduction

According to the World Health Organization (WHO), non-communicable diseases (NCDs) are chronic diseases which tend to have long duration. The four major types of NCDs include cardiovascular conditions, cancers, chronic respiratory diseases and diabetes [1]. Type 2 diabetes is a serious and chronic disease which develops when the body cannot use insulin properly [2]. The majority (≥ 95%) of people with diabetes are affected by type 2 diabetes [2, 3]. Globally, from 1990 to 2017, the age-standardized prevalence rates of type 2 diabetes has increased from 4,576.7 to 5722.1 [4]. This increase is attributable to the increase of associated risk factors such as being overweight or obese [2].

Referring to the World Bank Income Level divisions, the prevalence of type 2 diabetes has increased substantially in all regions. Nevertheless, all regions except the high-income regions reported an increase of mortality due to type 2 diabetes, particularly in the lower-middle-income region [4]. In Malaysia, the population living with type 2 diabetes has increased from 11.2% in 2011 to 13.4% in 2015, and 18.3% in 2019 [5]. Diabetes was also reported to be the sixth largest contributor of deaths among Malaysians [6].

The global economic burden of type 2 diabetes is substantial, with the estimated global health expenditure of US$760 billion. This figure is projected to increase to US$845 billion by the year 2045 [7]. Additionally, the loss of productivity or ability to work caused by diabetes also pose serious threat to economic stability in many nations [8]. In Malaysia, the Disability-adjusted life years (DALYs) losses from cardiovascular disease, diabetes and cancer totalled RM 100.79 billion [9], while the estimated annual cost of type 2 diabetes was approximately US$600 million [10].

The WHO have identified four modifiable key behavioural risk factors for NCDs such as diabetes, namely unhealthy diet, harmful use of alcohol, tobacco use and physical inactivity [11]. According to the Health Belief Model (HBM), health-related behaviours are determined by risk perceptions (perceived susceptibility, perceived severity, perceived benefits, perceived barriers). The Health Belief Model posits that for a person the change his/her behaviours, he/she need to perceive the likelihood of developing a disease, concern about the severity of the disease, and perceives the benefit of taking action towards preventing the disease taking into consideration the perceived cost/barrier. Additionally, the model also suggests that cue to action or trigger is necessary for prompting health-related behaviour [12, 13].

In line with the HBM, several studies have shown that perceived risk of developing certain disease can affect an individual’s behaviour or attitude towards preventive health interventions. For example, a study found that patients with heart disease who had higher risk perception of having heart disease again in the future were more likely to adhere to medications and attend cardiac rehabilitation [14]. Similarly, a study conducted in the United States reported an association between high risk perception of breast cancer and the use of mammography for breast cancer screening [15]. Another study conducted in Malaysia reported that risk perception of type 2 diabetes was associated with participation in sufficient physical activity [16].

To address the increasing burden of type 2 diabetes in Malaysia, a better understanding on its risk perception is needed as a guide for preventive interventions. Research questionnaire is one of the primary methods used to assess the risk perception of type 2 diabetes [17, 18]. However, the psychometrics of the most widely used questionnaire [Risk Perception Survey for Developing Diabetes (RPS-DD)] are not well understood in racially diverse populations that tend to have higher prevalence of diabetes risk factors [19–22]. Thus, this questionnaire may not be applicable to the multiracial Malaysian population with high prevalence of diabetes risk factors [5, 23].

In Malaysia, the currently available questionnaire does not specifically assess risk perception of type 2 diabetes, but instead assesses risk perception of five common NCDs including hypertension, diabetes, heart disease, cancer and stroke [24]. Besides, it was not mentioned whether the questionnaire was developed based on any theory or model. Confirmatory factor analysis was used to validate the questionnaire, but the detailed analysis was not provided. The use of a validated questionnaire is important to ensure reliable and valid measurement [25]. Moreover, a robust questionnaire developed based on theory or model is needed in order to comprehensively measure the various dimensions of risk perception of type 2 diabetes [26].

To date, no Malay-language questionnaire has been developed specifically for assessing the risk perception of type 2 diabetes. Even though few questionnaires that assess risk perception of type 2 diabetes have been developed in countries outside Malaysia [27], a good questionnaire developed in one language or culture may not necessarily “travel well” across cultures due to differences in meaning and interpretation [28]. Therefore, the present study was aimed at developing and validating a Malay-language questionnaire (RPDM) to assess the risk perception of type 2 diabetes among Malaysians.

## Materials and methods

### Study design and population

This cross-sectional study was conducted from December 2022 to September 2023. The eligibility criteria were adults aged 18–59 years, have no diabetes and mental health illness. For pilot testing, adult respondents were recruited through WhatsApp which is among the most commonly used social media among Malaysians. The link to a google survey form is attached along with the WhatsApp invitation message. In the first section of the google survey form, the information regarding the purpose of the study was provided, followed by informed consent. Respondents who agree to participate and have their data used for the study may sign the online consent and have their data submitted online.

For subsequent validation, working adults from a tertiary institution in Malaysia were invited to participate in the study through their official email, where information about the study and the link to a google survey form were included. Informed consent was provided in the first section of the google survey form. Respondents who agree to participate and have their data used for the study may click the “I agree to take part in this study” button and have their data submitted online. Those who do not agree to participate may click the “I disagree to take part in this study” button and their data is not submitted and collected online. The online data collection was approved by the Universiti Kebangsaan Malaysia Research Ethics Committee.

### Questionnaire development

#### Item generation and formatting

To guide the development of the questionnaire items, a systematic review was carried out to identify previously developed questionnaire assessing risk perception of type 2 diabetes [27]. The conceptualization of constructs was based on the HBM in addition to previous literature. We adapted the questionnaire items from several studies [29–31] and developed a 59-item question pool. These items were to assess six constructs, namely i) perceived susceptibility (12 items), ii) perceived severity (10 items), iii) perceived benefit (9 items), iv) perceived barrier (10 items), v) cue to action (9 items), and vi) perceived self-efficacy (9 items).

For scoring of perceived susceptibility, perceived severity, perceived benefit, perceived barrier and cue to action, a 10-point Likert scale was used, where one is strongly disagree and 10 is strongly agree. For perceived self-efficacy, a 10-point Likert scale was used, where one is not at all confident and 10 is strongly confident.

#### Content validity and translation

Content validity was evaluated by four experts, including two Public Health Medicine Specialists, one Family Medicine Specialist and one Endocrinologist. The experts evaluated the questionnaire items in terms of their relevance on risk perception of type 2 diabetes, as well as their appropriateness in terms of simplicity, ambiguity, validity, and sentence structure. The questionnaire items were modified according to the feedback by the experts. Ambiguous questions were rephrased for clarity and unnecessary items were removed.

The modified questionnaire were then evaluated using the content validity index (CVI), where each item was rated using a 4-point Likert scale (1 = not relevant, 2 = unable to assess relevance without item revision, 3 = relevant, 4 = very relevant) [32]. The overall scale-CVI of ≥ 0.8 is considered as good [33]. The questionnaire underwent forward and backward translation by two independent translators, including a bilingual researcher and a qualified linguistic expert. The original English-language questionnaire was translated into Malay version with the original meaning being preserved.

### Questionnaire construct validation

#### Pre-test

To establish face validity, a pre-test was carried out among five adults to ensure comprehensibility and readability of the questionnaire.

#### Pilot-test

Following the pre-test, a pilot testing was conducted among 100 respondents to assess the construct validity and reliability of the questionnaire. Construct validity was tested using exploratory factor analysis (EFA). EFA generally requires larger samples, with a minimum of 50 respondents [34]. This study followed the recommendation by Hair et al. to use at least 100 respondents for factor analysis [35]. A convenience sampling method was used to recruit respondents.

The factor extraction was performed using Principal Component Analysis (PCA) and varimax rotation method. Determination of retained factors was based on Kaiser criterion with eigenvalues greater than one, while determination of retained items were based on factor loading of ≥ 0.40. A Kaiser-Meyer-Olkin (KMO) value of > 0.50 indicated adequate sample size, while a significant Bartlett’s value of < 0.05 was accepted for sphericity valuation test [36]. Cronbach’s alpha (α) was used to assess the reliability of each construct.

#### Subsequent validation

For subsequent validation, data was collected from 158 respondents. This sample size was based on the recommendation by Hair et al. that factor analysis requires at least 100 samples [35]. A convenience sampling method was used to recruit respondents. To statistically test the EFA proposed hypothesis of risk perception of type 2 diabetes, a confirmatory factor analysis (CFA) was carried out using item parceling. Parceling is a common technique used in CFA and Structural Equation Modeling (SEM) when at least two items are combined (summed or average) before conducting an analysis and the parcels are used as the manifest indicators of latent constructs [37]. Parceling produces composite items with improved variance which are more congruent for factor analysis [38].

SEM technique was used to assess the model fit measures, constructs’ convergent validity, reliability and discriminant validity. Several model fit indicators were used, including relative chi-square (χ2/df < 3), root mean square error of approximation (RMSEA < 0.08) [39], comparative fit index (CFI > 0.9) [40], goodness of fit index (GFI > 0.9) [41] and normed fit index (NFI > 0.9) [42]. Convergent validity is met if the average variance extracted (AVE) is more or equal to 0.5 [43], while construct reliability of more or equal to 0.7 is acceptable [39]. The discriminant validity is satisfied if square correlation values of one construct with other constructs are less than the AVE of the specific construct [44].

### Statistical analysis

The data were analysed using the Statistical Packages for the Social Sciences (SPSS) version 21 and SPSS AMOS version 28. Descriptive statistics of frequencies (*n*) and percentages (%) were used for qualitative data, while the appropriate measure of central tendency was used for quantitative data.

### Ethical approval

Ethical approval for the study was obtained from the Universiti Kebangsaan Malaysia Research Ethics Committee (FF-2022-286) prior to commencement of the study.

## Results

### Study population

A total of 100 respondents with the median age of 35.00 years (IQR = 29.00–40.75) participated in the pilot test (Table 1). Majority of the respondents were women (68.0%), aged 30–39 years (47.0%), Malay (56.0%), and had tertiary education (91.0%). As shown in Table 1, the subsequent validation study had 158 respondents with the median age of 45.00 years (IQR = 42.00–50.00). Majority of the respondents were women (67.1%), aged 40–49 years (59.5%), Malay (96.2%), and had tertiary education (81.6%).

**Table 1:** Characteristics of respondents.

Factors	Pilot test (n = 100)	Subsequent validation (n = 158)
	*n* (%)	*n* (%)
**Sociodemographic characteristics**		
**Gender**		
Male	32 (32.0)	52 (32.9)
Female	68 (68.0)	106 (67.1)
**Age (years)**	Median (IQR): 35.00 (29.00–40.75)	Median (IQR): 45.00 (42.00–50.00)
18–29	26 (26.0)	1 (0.6)
30–39	47 (47.0)	17 (10.8)
40–49	17 (17.0)	94 (59.5)
50–59	10 (10.0)	46 (29.1)
**Ethnicity**		
Malay	56 (56.0)	152 (96.2)
Chinese	34 (34.0)	4 (2.5)
Indian	3 (3.0)	-
Others	7 (7.0)	2 (1.3)
**Education level**		
Secondary school/ less	9 (9.0)	29 (18.4)
College or university	91 (91.0)	129 (81.6)

Abbreviation: IQR–Interquartile range

### Questionnaire development and construct validation

Based on the suggestions by the four experts, 55 items were retained after reduction and modification of items to ensure readability and explicitness. The scale-CVI of the 55-item Malay version RPDM was 0.82. To obtain face validity, some modification was made according to the suggestions provided by the respondents in pre-test.

### Pilot test

Before EFA, KMO measure of sampling adequacy and Bartlett’s test of sphericity assumptions were tested. The KMO value was 0.696, while the Bartlett’s test of sphericity was significant (Table 2). Based on the eigenvalue of ≥ one, 13 factors were generated initially. However, the items representing five constructs failed to be theoretically meaningful according to the HBM, while two constructs had < 3 items with a loading ≥ 0.4 [45]. Thus, the outcome was interpreted as six constructs in accordance with the HBM and previous literature [12, 30, 31].

**Table 2:** Kaiser-Meyer-Olkin and Bartlett’s test and total variance explained.

Kaiser-Meyer-Olkin Measure of Sampling Adequacy		0.696
Bartlett’s test of sphericity	Approx. chi-square	4212.89
	Df	1485
	Sig.	<0.001

Four items with low factor loadings (<0.40), two cross-loading items and one item that did not load on the intended factor were removed. The cross-loading items were removed one by one, starting from the item that gives a high load on two factors with the smallest difference between factors loadings until no cross-loading items remain [46]. During the analysis, in order that each factor had at least three items with factor loadings ≥ 0.4, the number of factors was reduced to five.

Table 3 shows the findings of the pilot study. After the above mentioned steps, a total of 48 items remained. There were cue to action (7 items) and perceived self-efficacy (9 items) in construct 1, perceived severity (9 items) in construct 2, perceived benefit (7 items) in construct 3, perceived susceptibility (8 items) in construct 4 and perceived barrier (8 items) in construct 5. The five constructs were named according to the loading items, i.e., perceived self-efficacy (Peff) for construct 1, perceived severity (Psev) for construct 2, perceived benefit (Pbnf) for construct 3, perceived susceptibility (Psus) for construct 4 and perceived barrier (Pbar) for construct 5.

**Table 3:** Pilot study for RPDM constructs.

Item	Mean (SD)	Component and factor loading					Reliability analysis	
		1	2	3	4	5	CITC	Cronbach’s α
Cue_1	5.56 (2.10)	0.52					0.55	0.93
Cue_2	5.05 (2.30)	0.54					0.56	
Cue_3	5.68 (2.75)	0.61					0.58	
Cue_4	6.02 (2.35)	0.51					0.59	
Cue_5	7.04 (3.13)	0.47					0.44	
Cue_6	8.36 (1.85)	0.60					0.61	
Cue_7	8.03 (2.05)	0.66					0.67	
Peff_1	7.35 (2.08)	0.79					0.74	
Peff_2	7.03 (2.21)	0.58					0.53	
Peff_3	6.84 (2.14)	0.76					0.75	
Peff_4	7.14 (2.17)	0.80					0.76	
Peff_5	7.26 (2.02)	0.77					0.75	
Peff_6	7.17 (2.23)	0.78					0.73	
Peff_7	6.92 (2.18)	0.84					0.78	
Peff_8	6.12 (2.21)	0.72					0.68	
Peff_9	6.91 (2.00)	0.76					0.72	
Psev_2	8.28 (2.06)		0.81				0.71	0.90
Psev_3	8.43 (1.83)		0.80				0.74	
Psev_4	7.91 (2.05)		0.79				0.70	
Psev_5	7.55 (2.35)		0.80				0.72	
Psev_6	6.75 (2.80)		0.81				0.74	
Psev_7	7.51 (2.72)		0.75				0.73	
Psev_8	7.12 (2.89)		0.66				0.61	
Psev_9	8.20 (2.33)		0.43				0.48	
Psev_12	7.32 (2.71)		0.54				0.59	
Pbnf_1	9.03 (2.03)			0.72			0.70	0.91
Pbnf_2	8.91 (2.12)			0.86			0.80	
Pbnf_3	8.23 (2.33)			0.81			0.83	
Pbnf_5	8.05 (2.29)			0.82			0.81	
Pbnf_6	8.59 (2.08)			0.87			0.79	
Pbnf_7	7.24 (2.55)			0.51			0.58	
Pbnf_8	8.87 (1.88)			0.71			0.69	
Psus_1	8.67 (2.16)				0.55		0.39	0.81
Psus_2	5.23 (2.99)				0.68		0.57	
Psus_3	3.12 (2.08)				0.44		0.30	
Psus_5	7.22 (2.85)				0.72		0.61	
Psus_6	6.45 (2.77)				0.64		0.64	
Psus_7	4.71 (2.94)				0.48		0.43	
Psus_8	8.65 (2.13)				0.68		0.64	
Psus_9	7.60 (2.53)				0.63		0.59	
Pbar_1	3.06 (2.53)					0.61	0.50	0.77
Pbar_2	3.97 (2.75)					0.48	0.32	
Pbar_3	4.88 (3.06)					0.62	0.49	
Pbar_4	4.77 (2.84)					0.55	0.57	
Pbar_5	4.30 (2.75)					0.62	0.50	
Pbar_6	5.45 (2.78)					0.66	0.52	
Pbar_7	2.47 (2.08)					0.49	0.36	
Pbar_10	3.64 (2.58)					0.54	0.48	

Abbreviation: CITC–Corrected item total correlation

The five-factor model of EFA accounted for 55.18% of the total variance. The constructs’ reliability were assessed using Cronbach’s α and the values were between 0.77 to 0.93 for all constructs. S1 Table summarises the details of the corresponding items and statements of the diabetes risk perception constructs.

### Subsequent validation

A CFA of the 48 item five-factor model was conducted using item parceling. Two parcels comprised of nine and seven items were generated for the perceived self-efficacy construct. In view of the multidimensional nature of the construct, the facet-representative parceling approach was used, where items that share secondary facet-relevant content are combined into the same parcel [38]. Three parcels each were generated for the perceived benefit, perceived susceptibility and perceived barrier constructs. The item-to-construct balance approach was used for the perceived benefit and perceived susceptibility constructs. This approach is based on a goal of using parcels that are equally balanced in terms of their difficulty and discrimination [38].

Item paceling for the perceived barrier construct was carried out using the correlated uniqueness approach. This approach uses modification indices to determine which items have uniqueness that covary and combine these items into the same parcel [47]. The nine items of the perceived severity construct did not require parceling. The model fit indices of the perceived severity construct were improved by correlating items with high modification indices using double-headed arrows.

**Fig 1:**
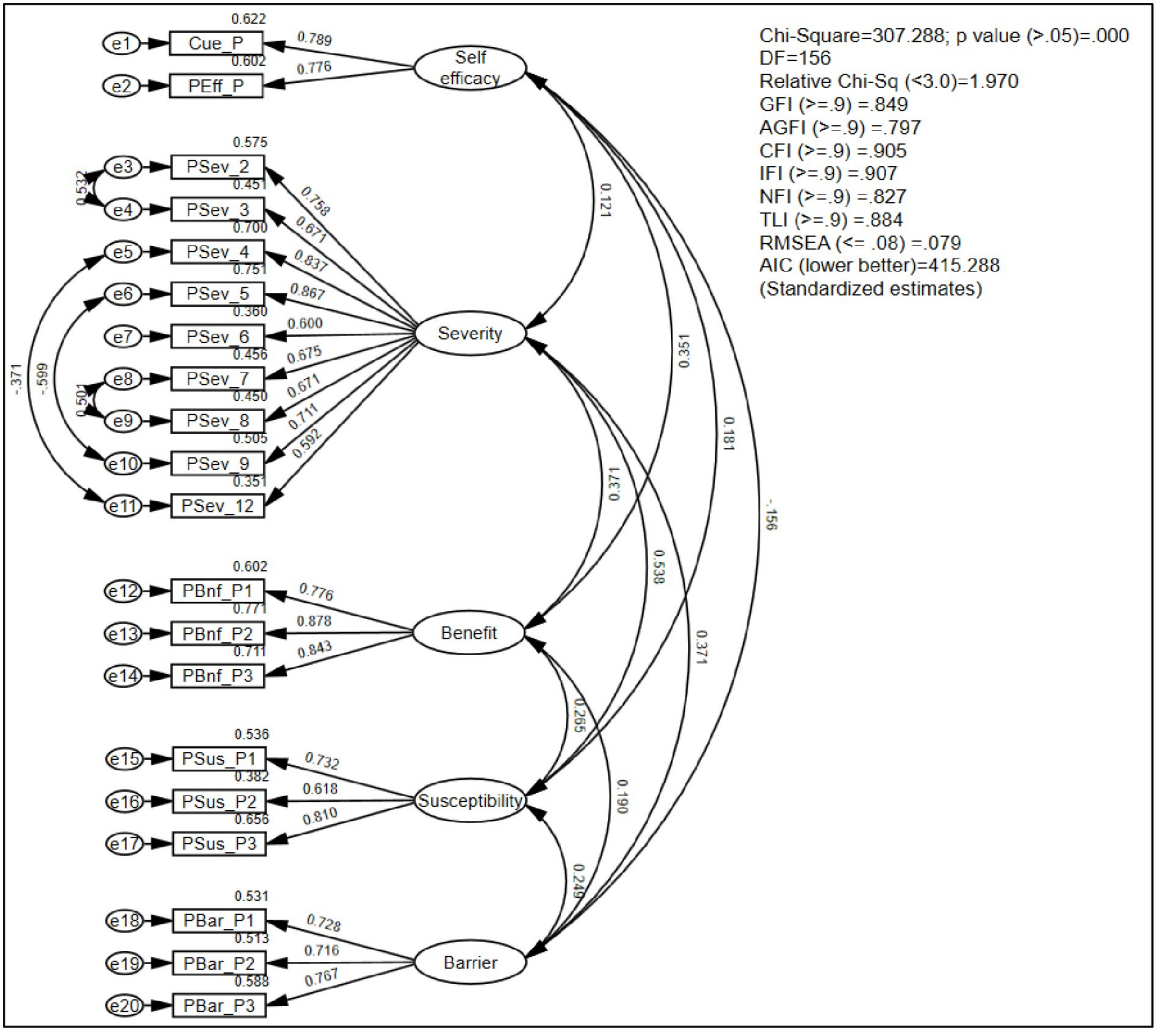
CFA of the diabetes risk perception model.

The CFA five-factor model (RPDM) was described using the SEM diagram (Fig 1). All latent variables were described in ellipses, while all manifest variables were presented in rectangles. Double-headed arrows represented correlations and single-headed arrows represented standardized loading between manifest and latent variables. Table 4 shows the factor loading of each item/parcel with their respective construct while Table 5 shows CFA result for the measurement model.

**Table 4:** Factor loading of each item/parcel with their respective construct.

Construct	Item/ Parcel	Factor Loading
Perceived self-efficacy (Peff)	Cue parcel (item cue_1, cue_2, cue_3, cue_4, cue_5, cue_6, cue_7)	0.79
	Perceived-efficacy parcel (item Peff1, Peff2, Peff3, Peff 4, Peff5, Peff6, Peff7, Peff8, Peff9)	0.78
Perceived severity (Psev)	Psev_2	0.76
	Psev_3	0.67
	Psev_4	0.84
	Psev_5	0.87
	Psev_6	0.60
	Psev_7	0.68
	Psev_8	0.67
	Psev_9	0.71
	Psev_12	0.59
Perceived Benefit (Pbnf)	Perceived benefit parcel 1 (item Pbnf_1, Pbnf_6, Pbnf_7)	0.78
	Perceived benefit parcel 2 (item Pbnf_2, Pbnf_3)	0.88
	Perceived benefit parcel 3 (item Pbnf_5, Pbnf_8)	0.84
Perceived susceptibility (Psus)	Perceived susceptibility parcel 1 (item Psus_3, Psus_5, Psus_7)	0.73
	Perceived susceptibility parcel 2 (item Psus_1, Psus_2, Psus_8)	0.62
	Perceived susceptibility parcel 3 (item Psus_6, Psus_9)	0.81
Perceived barrier (Pbar)	Perceived barrier parcel 1 (item Pbar_1, Pbar_3, Pbar_5)	0.73
	Perceived barrier parcel 2 (item Pbar_2, Pbar_6, Pbar_10)	0.72
	Perceived barrier parcel 3 (item Pbar_4, Pbar_7)	0.77

**Table 5:** Construct reliability, average variance extracted (on the diagonal) and squared correlation coefficients (on the off-diagonal).

Construct	Construct reliability	Peff	Psev	Pbnf	Psus	Pbar
Peff	0.760	**0.612**				
Psev	0.902	0.015	**0.511**			
Pbnf	0.872	0.123	0.138	**0.695**		
Psus	0.766	0.033	0.289	0.070	**0.525**	
Pbar	0.876	0.024	0.138	0.036	0.062	**0.702**

**Fig 2:**
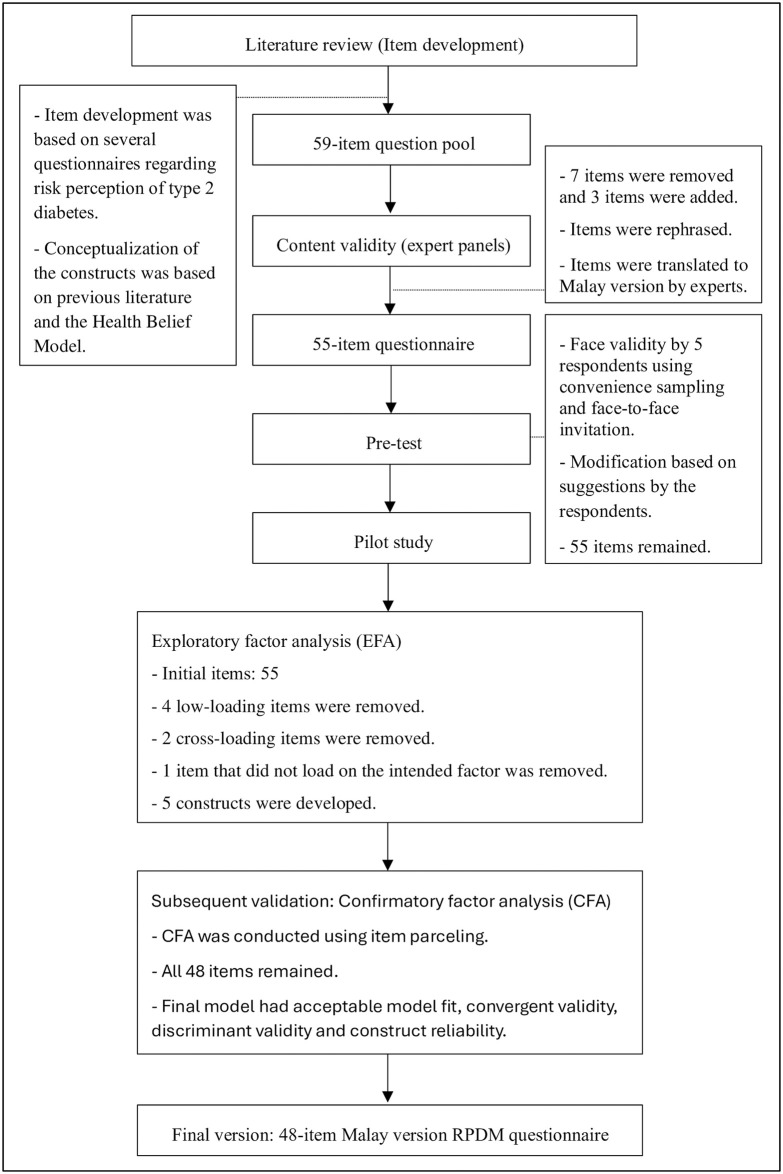
Flowchart of the development and validation of the Malay version RPDM questionnaire.

The convergent validity and discriminant validity of RPDM were assessed based on the AVE values. The AVE values for all the five constructs were > 0.5. All square correlation values were lesser than the AVE values of their respective constructs. The construct reliability values of the five constructs ranged between 0.76 and 0.90. The five-factor model had an acceptable model fit, i.e., χ2/df < 3, RMSEA < 0.08 and CFI = 0.905. Thus, CFA proved that the risk perception of diabetes had five latent constructs. The overall results suggest that the RPDM has fair psychometric properties and acceptable model fit. Fig 2 summarises the process of the questionnaire development and validation.

## Discussion

This study aimed to develop a questionnaire which can assess the risk perception of type 2 diabetes among Malaysians. The guidelines for questionnaire development and validation was followed–identification of construct of interest, questionnaire development and questionnaire validation [48]. A 59-item question pool was generated initially. The overall scale-CVI of ≥ 0.8 ensured the content validity of the 55-item questionnaire after reduction and modification [33], while face validity was obtained through the pre-test.

This questionnaire adequately and appropriately covered the multiple dimensions of risk perception of type 2 diabetes (perceived self-efficacy, perceived severity, perceived benefit, perceived susceptibility, and perceived barrier) based on the HBM [12, 13] and previous literature [29–31]. This questionnaire and a questionnaire previously developed in Myanmar shared similar dimensions except for the perceived severity dimension, which was dropped in the later questionnaire [30]. One explanation is that Myanmar people might perceive severity of NCDs differently due to various contextual and cultural factors [30]. The KMO value of 0.696 indicated adequate sample size, while the significant Bartlett’s test of sphericity indicated that the variances are not equal across groups [36]. The total variance explained of 55.18% is considered acceptable [45].

During the EFA, a total of seven items were removed, with the perceived severity construct having the highest number of removed items. For the perceived severity construct, two items were removed due to low factor loading (Psev_10, Psev_11), and one item was removed due to cross-loading between factors (Psev_1). Item Psev_10 (diabetes is a disease that cannot be cured at all) and item Psev_11 (managing diabetes requires a lot of money) had no intrinsic ability to capture diabetes severity perception. This may be due to that many Malaysians wrongly believe that diabetes can be cured [49], and public healthcare services in Malaysia are heavily subsidized with very low user fee [50].

Item Psev_1 (diabetes can cause a serious health problem to me) cross-loads onto other factor, which may be due to the content of the item not being fully understood, relevant, or meaningful. Apart from that, the respondents had no known diabetes hence might perceive the severity of diabetes differently. Moreover, the serious complications of diabetes are only apparent over time and therefore could also affect the perception of severity among the respondents [51].

For the perceived barrier construct, item Pbar_9 (on most days of a week, I don’t have time to do 30 minutes exercise a day) was removed due to low factor loading, and item Pbar_8 (I don’t know about the suitable physical exercises to prevent diabetes) was removed due to cross-loading between factors. In item Pbar_9, the phrase “on most days of a week” is rather vague which may have resulted in respondents rejecting it as a legitimate item. The cross-loading of item Pbar_8 may have resulted from ambiguity in content, as ambiguous wording may introduce construct-irrelevant variance into the scores [52].

For the perceived susceptibility construct, item Psus_4 (there is possibility that I have diabetes at this moment) was removed due to low factor loading. According to a national survey, a large proportion of Malaysians (about three-fifths) had screening for NCDs including diabetes in the recent months [53]. As a result, many respondents may accurately know whether they have diabetes at present, which may affect their responses on this item. For the perceived benefit construct, item Pbnf_4 (being a non-smoker can reduce the risk of having diabetes) was removed as it did not load on the intended factor. Respondents may respond to this item differently, as studies have shown that about half of Malaysians failed to identify smoking as a risk factor for diabetes [54], and only one-third of Malaysians agreed that quitting smoking can reduce the risk of diabetes [55].

As the EFA yielded a five-factor model, one construct (cue to action) could not form an independent construct. Construct 1 which contained both the cue to action and perceived self-efficacy items was named as perceived self-efficacy (Peff) as the perceived self-efficacy dimension dominates with more items. Previous study also adopted this concept where a multidimensional construct was named based on the dimension that predominates [30]. In previous study, cue to action and perceived self-efficacy were conceptualized as two separate dimensions in a questionnaire assessing risk perception of NCDs. However, the questionnaire was only assessed for content validity, while construct validity was not assessed [31].

A meta-analysis study reported that perceived barrier was the most powerful dimension of the HBM, followed by perceived benefit, perceived susceptibility and perceived severity [56]. Even though the HBM includes cue to action, but this dimension is the most underdeveloped and rarely researched. Self-efficacy was also proposed as a HBM variable but it is understudied [57]. Despite being understudied, self-efficacy has been consistently identified as a strong predictor of preventive behaviours towards diabetes [58–60].

A CFA was carried out using item parceling. Besides the advantage of requiring smaller sample size, item parceling also results in better reliability and improved model fit [61]. CFA demonstrated that the five-factor model of RPDM has an acceptable model fit. The AVE values of the five constructs ranged between 0.51 to 0.70. These AVE values were above the cut-off value of 0.5, indicating sufficient convergent validity [39]. There was sufficient discriminant validity as all the square correlation values were lower that the AVE values of their respective factors [44]. All the five constructs had construct reliability of > 0.7, suggesting that all constructs had adequate internal consistency [39]. Similarly, a five-factor questionnaire designed to assess risk perception of NCDs based on the HBM, also demonstrated good model fit, as well as satisfactory convergent validity, discriminant validity, and construct reliability [30].

## Strengths and limitations

To the best of the authors’ knowledge, this is among the first study in Malaysia that developed and validated a Malay-version risk perception questionnaire on type 2 diabetes (RPDM). Even though the sample size of 100 for the pilot test is just adequate for EFA [35], the strength of this study is the inclusion of large sample size (158) for subsequent validation study. Hair et al. suggested that the minimum sample size is 100 for model containing five or fewer constructs [39], while the other suggested to have a minimum of five times as many observations as the number of variables to be analyzed (i.e., 240 respondents for a 48-item questionnaire) [62]. Nevertheless, item parceling approach yielded 20 parameters hence only 100 observations are required [61].

This study is not without limitation. Convenience sampling was used in this study which may have caused sampling bias and limited the generalizability of our findings. Because the sociodemographic characteristics differ between our study and the general population, it is possible that the results are only partially generalizable to the larger Malaysian population [63]. Nevertheless, previous studies in Malaysia have proven that the level of diabetes risk perception did not differ significantly across sociodemographic (gender, age, ethnicity and education level) groups [64, 65]. The criterion validity was not assessed as there is currently no standardized tool available to use as a golden standard to assess risk perception of type 2 diabetes.

## Conclusion

The Malay version RPDM showed good psychometric properties. The questionnaire was successfully validated and may serve as a useful tool to measure the five constructs (perceived self-efficacy, perceived severity, perceived benefit, perceived susceptibility and perceived barrier) of risk perception of type 2 diabetes among Malaysians. Besides contributing to a better understanding of risk perception of type 2 diabetes among Malaysians, this Malay-language questionnaire can also be helpful to enhance diabetes prevention communication between healthcare providers and their clients. Further large-scale study is warranted to confirm the reliability and validity of the questionnaire. Future development work on the RPDM should take into account sociocultural and healthcare system variations in Malaysia.

## Supporting information

S1 Table Details of the corresponding items and statements of the diabetes risk perception construct. (DOCX)

S2 Table Validated final 48-item Malay version RPDM questionnaire. (DOCX)
