# Supplementary material for: Development and validation of a Malay version questionnaire for assessing risk perception of type 2 diabetes (RPDM)
Source: PLoS One. 2025 Jan 7;20(1):e0311834. doi: 10.1371/journal.pone.0311834 (PMC11706471; doi:10.1371/journal.pone.0311834)
Supplement: S1 Table — (DOCX) [file pone.0311834.s001.docx]

**S1 Table. Details of the corresponding items and statements of the diabetes risk perception construct.**

| **Construct** | **Item** | **Statement** |
| --- | --- | --- |
| Perceived self-efficacy | Cue_1* | Saya telah memakan diet yang sihat.  *I have been eating a healthy diet.* |
|  | Cue_2* | Saya telah mengelakkan makanan berlemak.  *I have been avoiding fatty foods.* |
|  | Cue_3* | Saya telah makan makanan dalam hidangan kecil.  *I have been eating small-portion meals.* |
|  | Cue_4* | Saya telah mengikuti nasihat perubatan sebagai manfaat kepada kesihatan saya.  *I have been following medical advice to benefit my health.* |
|  | Cue_5* | Jika saya ketagih merokok, saya akan mengurangkan risiko diabetes dengan cuba berhenti merokok.  *If I am addicted to smoking, I will reduce my risk of diabetes by trying to quit smoking.* |
|  | Cue_6* | Saya akan cuba mengekalkan berat badan yang sesuai untuk mencegah diabetes.  *I will try to maintain an appropriate body weight to prevent diabetes.* |
|  | Cue_7* | Saya akan cuba melakukan senaman berkala secara aktif untuk mencegah diabetes.  *I will try to do regular exercise actively to prevent diabetes.* |
|  | Peff_1* | Sejauh manakah keyakinan anda dalam mencegah diabetes?  *How confident are you in preventing diabetes?* |
|  | Peff_2* | Sejauh manakah anda yakin, anda boleh menghadiri pemeriksaan kesihatan secara berkala untuk mengesan diabetes lebih awal?  *How confident are you that you could attend regular health check-ups to detect diabetes early?* |
|  | Peff_3* | Sejauh manakah anda yakin, anda tahu cara mencegah diabetes?  *How confident are you that you know how to prevent diabetes?* |
|  | Peff_4* | Sejauh manakah anda yakin, terdapat banyak perkara boleh anda lakukan untuk mengurangkan peluang anda mendapat diabetes?  *How confident are you that there is a lot you can do to reduce your chances of getting diabetes?* |
|  | Peff_5* | Sejauh manakah anda yakin, anda boleh hidup dengan sihat untuk mencegah diabetes?  *How confident are you that you could live healthily to prevent diabetes?* |
|  | Peff_6* | Sejauh manakah anda yakin, anda boleh mengekalkan berat badan yang sesuai dengan bersenam secara berkala untuk mencegah diabetes?  *How confident are you that you could maintain appropriate body weight by exercising regularly to prevent diabetes?* |
|  | Peff_7* | Sejauh manakah anda yakin, anda boleh mengekalkan berat badan yang sesuai dengan memakan diet yang sihat untuk mencegah diabetes?  *How confident are you that you could maintain appropriate body weight by eating a healthy diet to prevent diabetes?* |
|  | Peff_8* | Sejauh manakah anda yakin, anda boleh hanya makan diet yang sihat pada kebanyakan hari?  *How confident are you that you could only eat a healthy diet on most days?* |
|  | Peff_9* | Sejauh manakah anda yakin, anda boleh mengurangkan risiko diabetes?  *How confident are you that you could reduce the risk of diabetes?* |
| Perceived severity | Psev_1 | Diabetes boleh menyebabkan masalah kesihatan yang serius kepada saya.  *Diabetes can cause a serious health problem to me.* |
|  | Psev_2* | Diabetes boleh menjejaskan kehidupan sosial saya.  *Diabetes can affect my social life.* |
|  | Psev_3* | Menghidap diabetes akan memberi kesan yang ketara kepada hidup saya.  *Having diabetes will have significant effects on my life.* |
|  | Psev_4* | Menghidap diabetes akan memberi kesan yang ketara kepada keluarga saya.  *Having diabetes will have significant effects on my family.* |
|  | Psev_5* | Menghidap diabetes akan memberi kesan yang ketara kepada kerja saya.  *Having diabetes will have significant effects on my work.* |
|  | Psev_6* | Menghidap diabetes akan memberi kesan yang ketara kepada pendapatan saya.  *Having diabetes will have significant effects on my income.* |
|  | Psev_7* | Menghidap diabetes boleh merosakkan diri saya.  *Having diabetes can ruin me.* |
|  | Psev_8* | Penampilan fizikal saya boleh terjejas jika saya menghidap diabetes.  *My physical appearance could be affected if I have diabetes.* |
|  | Psev_9* | Idea di mana saya menghidap diabetes, menakutkan saya.  *The idea of having diabetes scares me.* |
|  | Psev_10 | Diabetes adalah penyakit yang tidak boleh disembuhkan.  *Diabetes is a disease that cannot be cured at all.* |
|  | Psev_11 | Mengurus diabetes memerlukan banyak wang.  *Managing diabetes requires a lot of money.* |
|  | Psev_12* | Menghidap diabetes boleh membunuh saya.  *Having diabetes can kill me.* |
| Perceived benefit | Pbnf_1* | Kehidupan saya akan menjadi lebih baik jika saya tidak menghidap diabetes.  *My life will be better if I do not have diabetes.* |
|  | Pbnf_2* | Gaya hidup sihat boleh mencegah diabetes.  *A healthy lifestyle can prevent diabetes.* |
|  | Pbnf_3* | Mencapai berat badan yang sesuai boleh mencegah diabetes.  *Achieving an appropriate body weight can prevent diabetes.* |
|  | Pbnf_4 | Menjadi bukan perokok boleh mengurangkan risiko menghidap diabetes.  *Being a non-smoker can reduce the risk of having diabetes.* |
|  | Pbnf_5* | Melakukan senaman secara berkala boleh mencegah diabetes.  *Doing regular exercises can prevent diabetes.* |
|  | Pbnf_6* | Memakan diet yang sihat boleh mencegah diabetes.  *Eating a healthy diet can prevent diabetes.* |
|  | Pbnf_7* | Hidup tanpa tekanan boleh mencegah diabetes.  *Living stress-free can prevent diabetes.* |
|  | Pbnf_8* | Pemeriksaan kesihatan secara berkala diperlukan untuk pengesanan awal diabetes.  *A regular health check-up is necessary to detect diabetes early.* |
| Perceived susceptibility | Psus_1* | Diabetes adalah penyakit untuk orang tua.  *Diabetes is a disease for old people.* |
|  | Psus_2* | Saya berisiko menghidap diabetes.  *I am at risk of having diabetes.* |
|  | Psus_3* | Saya hampir pasti, akhirnya saya akan menghidap diabetes.  *I am almost sure that I will eventually have diabetes.* |
|  | Psus_4 | Saya berkemungkinan menghidap diabetes pada masa ini.  *There is possibility that I have diabetes at this moment.* |
|  | Psus_5* | Menjadi obes/berat badan berlebihan boleh menyebabkan saya menghidap diabetes.  *Being obese/overweight can lead me to have diabetes.* |
|  | Psus_6* | Jika keluarga saya menghidap diabetes, saya mungkin akan menghidapnya juga.  *If my family has diabetes, I will likely get it too.* |
|  | Psus_7* | Jika saya merokok, saya mungkin akan menghidap diabetes.  *If I smoke, I will likely get diabetes.* |
|  | Psus_8* | Tabiat pemakanan tidak sihat boleh menyebabkan saya menghidap diabetes.  *Unhealthy eating habits can lead me to have diabetes.* |
|  | Psus_9* | Kurang aktiviti fizikal boleh menyebabkan saya menghidap diabetes.  *Physical inactivity can lead me to have diabetes.* |
| Perceived barrier | Pbar_1* | Tidak banyak yang boleh saya lakukan untuk mencegah diabetes.  *There is nothing much I can do to prevent diabetes.* |
|  | Pbar_2* | Tiada rawatan berkesan untuk menyembuhkan diabetes.  *There is no effective treatment to cure diabetes.* |
|  | Pbar_3* | Makan makanan sihat memerlukan banyak wang.  *Eating healthy foods requires a lot of money.* |
|  | Pbar_4* | Adalah mahal untuk melakukan pemeriksaan kesihatan secara berkala.  *It is expensive to have regular health check-ups.* |
|  | Pbar_5* | Menyediakan makanan sihat mengambil masa.  *Preparing healthy foods is time-consuming.* |
|  | Pbar_6* | Pemeriksaan kesihatan mengambil masa.  *Health check-ups are time-consuming.* |
|  | Pbar_7* | Saya berasa malu untuk pergi membuat pemeriksaan kesihatan.  *I feel embarrassed to go for health check-ups.* |
|  | Pbar_8 | Saya tidak tahu senaman fizikal yang sesuai untuk mencegah diabetes.  *I don’t know about the suitable physical exercises to prevent diabetes.* |
|  | Pbar_9 | Pada kebanyakan hari dalam seminggu, saya tidak mempunyai masa untuk bersenam 30 minit sehari.  *On most days of a week, I don’t have time to do 30 minutes exercise a day.* |
|  | Pbar_10* | Saya tidak tahu jenis diet yang boleh mencegah diabetes.  *I don’t know which type of diet can prevent diabetes.* |

*Finalized items
