# Supplementary material for: Development and validation of a Malay version questionnaire for assessing risk perception of type 2 diabetes (RPDM)
Source: PLoS One. 2025 Jan 7;20(1):e0311834. doi: 10.1371/journal.pone.0311834 (PMC11706471; doi:10.1371/journal.pone.0311834)
Supplement: S2 Table — (DOCX) [file pone.0311834.s002.docx]

**S2 Table. Validated final 48-item Malay version RPDM questionnaire.**

| **Item** | **Pernyataan** | **Pengukuran** | | | | | | | | | |
| --- | --- | --- | --- | --- | --- | --- | --- | --- | --- | --- | --- |
| **Persepsi kerentanan** | | **Sangat tidak setuju 🡨🡪 Sangat bersetuju** | | | | | | | | | |
| **Psus_1** | Diabetes adalah penyakit untuk orang tua. | 1 | 2 | 3 | 4 | 5 | 6 | 7 | 8 | 9 | 10 |
| **Psus_2** | Saya berisiko menghidap diabetes. | 1 | 2 | 3 | 4 | 5 | 6 | 7 | 8 | 9 | 10 |
| **Psus_3** | Saya hampir pasti, akhirnya saya akan menghidap diabetes. | 1 | 2 | 3 | 4 | 5 | 6 | 7 | 8 | 9 | 10 |
| **Psus_5** | Menjadi obes/berat badan berlebihan boleh menyebabkan saya menghidap diabetes. | 1 | 2 | 3 | 4 | 5 | 6 | 7 | 8 | 9 | 10 |
| **Psus_6** | Jika keluarga saya menghidap diabetes, saya mungkin akan menghidapnya juga. | 1 | 2 | 3 | 4 | 5 | 6 | 7 | 8 | 9 | 10 |
| **Psus_7** | Jika saya merokok, saya mungkin akan menghidap diabetes. | 1 | 2 | 3 | 4 | 5 | 6 | 7 | 8 | 9 | 10 |
| **Psus_8** | Tabiat pemakanan tidak sihat boleh menyebabkan saya menghidap diabetes. | 1 | 2 | 3 | 4 | 5 | 6 | 7 | 8 | 9 | 10 |
| **Psus_9** | Kurang aktiviti fizikal boleh menyebabkan saya menghidap diabetes. | 1 | 2 | 3 | 4 | 5 | 6 | 7 | 8 | 9 | 10 |
| **Persepsi keterukan** | | **Sangat tidak setuju 🡨🡪 Sangat bersetuju** | | | | | | | | | |
| **Psev_2** | Diabetes boleh menjejaskan kehidupan sosial saya. | 1 | 2 | 3 | 4 | 5 | 6 | 7 | 8 | 9 | 10 |
| **Psev_3** | Menghidap diabetes akan memberi kesan yang ketara kepada hidup saya. | 1 | 2 | 3 | 4 | 5 | 6 | 7 | 8 | 9 | 10 |
| **Psev_4** | Menghidap diabetes akan memberi kesan yang ketara kepada keluarga saya. | 1 | 2 | 3 | 4 | 5 | 6 | 7 | 8 | 9 | 10 |
| **Psev_5** | Menghidap diabetes akan memberi kesan yang ketara kepada kerja saya. | 1 | 2 | 3 | 4 | 5 | 6 | 7 | 8 | 9 | 10 |
| **Psev_6** | Menghidap diabetes akan memberi kesan yang ketara kepada pendapatan saya. | 1 | 2 | 3 | 4 | 5 | 6 | 7 | 8 | 9 | 10 |
| **Psev_7** | Menghidap diabetes boleh merosakkan diri saya. | 1 | 2 | 3 | 4 | 5 | 6 | 7 | 8 | 9 | 10 |
| **Psev_8** | Penampilan fizikal saya boleh terjejas jika saya menghidap diabetes. | 1 | 2 | 3 | 4 | 5 | 6 | 7 | 8 | 9 | 10 |
| **Psev_9** | Idea di mana saya menghidap diabetes, menakutkan saya. | 1 | 2 | 3 | 4 | 5 | 6 | 7 | 8 | 9 | 10 |
| **Psev_12** | Menghidap diabetes boleh membunuh saya. | 1 | 2 | 3 | 4 | 5 | 6 | 7 | 8 | 9 | 10 |
| **Persepsi manfaat** | | **Sangat tidak setuju 🡨🡪 Sangat bersetuju** | | | | | | | | | |
| **Pbnf_1** | Kehidupan saya akan menjadi lebih baik jika saya tidak menghidap diabetes. | 1 | 2 | 3 | 4 | 5 | 6 | 7 | 8 | 9 | 10 |
| **Pbnf_2** | Gaya hidup sihat boleh mencegah diabetes. | 1 | 2 | 3 | 4 | 5 | 6 | 7 | 8 | 9 | 10 |
| **Pbnf_3** | Mencapai berat badan yang sesuai boleh mencegah diabetes. | 1 | 2 | 3 | 4 | 5 | 6 | 7 | 8 | 9 | 10 |
| **Pbnf_5** | Melakukan senaman secara berkala boleh mencegah diabetes. | 1 | 2 | 3 | 4 | 5 | 6 | 7 | 8 | 9 | 10 |
| **Pbnf_6** | Memakan diet yang sihat boleh mencegah diabetes. | 1 | 2 | 3 | 4 | 5 | 6 | 7 | 8 | 9 | 10 |
| **Pbnf_7** | Hidup tanpa tekanan boleh mencegah diabetes. | 1 | 2 | 3 | 4 | 5 | 6 | 7 | 8 | 9 | 10 |
| **Pbnf_8** | Pemeriksaan kesihatan secara berkala diperlukan untuk pengesanan awal diabetes. | 1 | 2 | 3 | 4 | 5 | 6 | 7 | 8 | 9 | 10 |
| **Persepsi halangan** | | **Sangat tidak setuju 🡨🡪 Sangat bersetuju** | | | | | | | | | |
| **Pbar_1** | Tidak banyak yang boleh saya lakukan untuk mencegah diabetes. | 1 | 2 | 3 | 4 | 5 | 6 | 7 | 8 | 9 | 10 |
| **Pbar_2** | Tiada rawatan berkesan untuk menyembuhkan diabetes. | 1 | 2 | 3 | 4 | 5 | 6 | 7 | 8 | 9 | 10 |
| **Pbar_3** | Makan makanan sihat memerlukan banyak wang. | 1 | 2 | 3 | 4 | 5 | 6 | 7 | 8 | 9 | 10 |
| **Pbar_4** | Adalah mahal untuk melakukan pemeriksaan kesihatan secara berkala. | 1 | 2 | 3 | 4 | 5 | 6 | 7 | 8 | 9 | 10 |
| **Pbar_5** | Menyediakan makanan sihat mengambil masa. | 1 | 2 | 3 | 4 | 5 | 6 | 7 | 8 | 9 | 10 |
| **Pbar_6** | Pemeriksaan kesihatan mengambil masa. | 1 | 2 | 3 | 4 | 5 | 6 | 7 | 8 | 9 | 10 |
| **Pbar_7** | Saya berasa malu untuk pergi membuat pemeriksaan kesihatan. | 1 | 2 | 3 | 4 | 5 | 6 | 7 | 8 | 9 | 10 |
| **Pbar_10** | Saya tidak tahu jenis diet yang boleh mencegah diabetes. | 1 | 2 | 3 | 4 | 5 | 6 | 7 | 8 | 9 | 10 |
| **Efikasi kendiri** | | **Sangat tidak setuju 🡨🡪 Sangat bersetuju** | | | | | | | | | |
| **Cue_1** | Saya telah memakan diet yang sihat. | 1 | 2 | 3 | 4 | 5 | 6 | 7 | 8 | 9 | 10 |
| **Cue_2** | Saya telah mengelakkan makanan berlemak. | 1 | 2 | 3 | 4 | 5 | 6 | 7 | 8 | 9 | 10 |
| **Cue_3** | Saya telah makan makanan dalam hidangan kecil. | 1 | 2 | 3 | 4 | 5 | 6 | 7 | 8 | 9 | 10 |
| **Cue_4** | Saya telah mengikuti nasihat perubatan sebagai manfaat kepada kesihatan saya. | 1 | 2 | 3 | 4 | 5 | 6 | 7 | 8 | 9 | 10 |
| **Cue_5** | Jika saya ketagih merokok, saya akan mengurangkan risiko diabetes dengan cuba berhenti merokok. | 1 | 2 | 3 | 4 | 5 | 6 | 7 | 8 | 9 | 10 |
| **Cue_6** | Saya akan cuba mengekalkan berat badan yang sesuai untuk mencegah diabetes. | 1 | 2 | 3 | 4 | 5 | 6 | 7 | 8 | 9 | 10 |
| **Cue_7** | Saya akan cuba melakukan senaman berkala secara aktif untuk mencegah diabetes. | 1 | 2 | 3 | 4 | 5 | 6 | 7 | 8 | 9 | 10 |
|  | | **Langsung tidak yakin 🡨🡪 Yakin sepenuhnya** | | | | | | | | | |
| **Peff_1** | Sejauh manakah keyakinan anda dalam mencegah diabetes? | 1 | 2 | 3 | 4 | 5 | 6 | 7 | 8 | 9 | 10 |
| **Peff_2** | Sejauh manakah anda yakin, anda boleh menghadiri pemeriksaan kesihatan secara berkala untuk mengesan diabetes lebih awal? | 1 | 2 | 3 | 4 | 5 | 6 | 7 | 8 | 9 | 10 |
| **Peff_3** | Sejauh manakah anda yakin, anda tahu cara mencegah diabetes? | 1 | 2 | 3 | 4 | 5 | 6 | 7 | 8 | 9 | 10 |
| **Peff_4** | Sejauh manakah anda yakin, terdapat banyak perkara boleh anda lakukan untuk mengurangkan peluang anda mendapat diabetes? | 1 | 2 | 3 | 4 | 5 | 6 | 7 | 8 | 9 | 10 |
| **Peff_5** | Sejauh manakah anda yakin, anda boleh hidup dengan sihat untuk mencegah diabetes? | 1 | 2 | 3 | 4 | 5 | 6 | 7 | 8 | 9 | 10 |
| **Peff_6** | Sejauh manakah anda yakin, anda boleh mengekalkan berat badan yang sesuai dengan bersenam secara berkala untuk mencegah diabetes? | 1 | 2 | 3 | 4 | 5 | 6 | 7 | 8 | 9 | 10 |
| **Peff_7** | Sejauh manakah anda yakin, anda boleh mengekalkan berat badan yang sesuai dengan memakan diet yang sihat untuk mencegah diabetes? | 1 | 2 | 3 | 4 | 5 | 6 | 7 | 8 | 9 | 10 |
| **Peff_8** | Sejauh manakah anda yakin, anda boleh hanya makan diet yang sihat pada kebanyakan hari? | 1 | 2 | 3 | 4 | 5 | 6 | 7 | 8 | 9 | 10 |
| **Peff_9** | Sejauh manakah anda yakin, anda boleh mengurangkan risiko diabetes? | 1 | 2 | 3 | 4 | 5 | 6 | 7 | 8 | 9 | 10 |
